# A Bi-Modulus Material Model for Bending Test on NHL3.5 Lime Mortar

**DOI:** 10.3390/ma16020486

**Published:** 2023-01-04

**Authors:** Rebecca Grazzini, Giulia Misseri, Luisa Rovero

**Affiliations:** Materials and Structures Division, Department of Architecture, University of Florence, Piazza Brunelleschi 6, 50121 Florence, Italy

**Keywords:** lime mortar, direct and compressive tensile test, bending tests, DIC, bi-modulus model, Timoshenko beam

## Abstract

The research provides an innovative contribution to the interpretation of three-point and four-point bending tests on mortars by employing a bi-modulus material model, which assumes an asymmetric constitutive law, i.e., different elastic moduli in tension and in compression. To this aim, Euler–Bernoulli and Timoshenko bi-modulus beam models are defined, and the related displacement fields are reported for three-point loading, and provided for the first time for the four-point bending layout. A wide experimental campaign, including uni-axial tensile and compressive tests, three-point and four-point bending tests, and on notched specimens three-point tests for mode-I fracture energy, has been carried out on lime mortar specimens exploiting traditional contact (CE-DT) and contactless (DIC) measurement systems. Experimental results provided the values of tensile and compressive mechanical characteristics, which are employed to validate estimations of the analytical model. The tension-to-compression moduli ratio experimentally observed is on average 0.52. Experimental outcomes of the DIC analysis proved the bi-modulus behaviour during the four-point bending tests showing visible shifting of the neutral axis. The bi-modulus analytical model provides closer results to the experimental ones for the slender specimens subjected to four-point bending.

## 1. Introduction

Characterisation of tensile properties of brittle construction materials constitutes a challenging task for the difficulties of implementing testing procedures that provide a sufficiently long-lasting uni-axial tensile stress state. Furthermore, since the tensile properties of brittle materials are related to micro-damage processes and fracture energy properties, specimen dimensions and loading rate might affect test results.

To estimate materials’ properties in tension, three-point bending tests [1] are often employed. In three-point bending tests, the stocky shape of the specimen and the concentrated loading condition might prevent a straightforward application of the classical beam theory, as highlighted in early experimental campaigns by Stokes [2] and reported by Love [3]. Nonetheless, the simplicity of the three-point bending test implementation motivates its broad application providing flexural strength according to the Bernoulli–Navier beam theory. The test pivots on the assumption of the same modulus in compression and in tension.

Experimental evidence confirms that the asymmetry of the tension-compression constitutive law is common to different materials, such as concrete [4], ceramics [5], rammed earth [6,7,8,9], graphite and composite materials [10,11], and biological materials [12,13], showing tension to compression moduli ratio ranging from values below the unit to orders of magnitude. No-tension and no-compression materials can also be analysed in the framework of bi-modulus theory, as shown in [14], where the tools of convex analysis are employed.

Bi-modulus materials have been already addressed by de Saint-Venant [15] considering the case of materials not following a linear constitutive law, for which it is assumed that stress distribution in a rectangular cross-section subject to pure bending can be described through to a polynomial law asymmetric with respect to the cross-section height. By further developing the same problem, Timoshenko [16] provides the shifted position of the neutral axis depending on the tension and compression Young’s moduli. In [17], the expression of a unique apparent Young’s modulus, depending on the compression and tension Young’s moduli and employed for the deflection estimation of a three-point bent beam is provided. In [18], this investigation is extended to the case of the four-point bending test. With Ambartsumyan [19] the bi-modulus material model is applied to isotropic plates and shells starting from the sign of principal stresses, imposing symmetry of the compliance matrix, $\nu_{t}/E_{t}=\nu_{c}/E_{c}$, and introducing a stress-sign-dependant shear modulus, which however does not ensure symmetry of the compliance matrix for all the directions. In [20], two weighting factors that preserve symmetry under rotation of the compliance, which changes according to the direction and magnitude of the principal stress state, are introduced.

In [21], endeavouring to fasten convergence problems in FE investigations, the authors define a shear modulus which preserves symmetry, can satisfy regression from principal to general reference system, is consistent with classical theory and depends on the magnitude, direction and sign of principal stresses. The latter approach is similarly employed by other authors [22,23]. In [24], two different exponential expressions representing the grade functions of tensile and compressive moduli of elasticity are assumed with a sign-independent Poisson’s ratio, so preserving the symmetry of the compliance. Some studies [6,7,8], apply the approach proposed in [21] to a micro-mechanical FE homogenization analysis for the definition of the critical surface of adobe brick masonry, assuming bi-modulus behaviour for mortar and blocks. Variational principles, which make use of internal variables to characterise the tension/compression state, have been defined as well in [4,25].

A constitutive bi-modulus model based on the definition of different stiffness matrices according to strain sign is provided in [11,26] with the aim of describing multi-layer composite laminates. In particular, one layer of a bi-modulus material shows the same bending-stretching coupling similar to a cross-ply laminate made of two layers [27]. Very few problems can be solved in a closed form if the asymmetry in the constitutive law is set, therefore, in order to solve 2D problems for bi-modulus materials, dedicated iterative algorithms must be calibrated, as thoroughly addressed in the review [28].

Concerning the bi-modulus beam model, in [29,30], the expression of the position of the neutral axis and then the displacement field for columns subject to compression and shear-free bending, and lateral force bending, are defined. In [31], the model proposed in [30] is employed to interpret test results of a wide experimental campaign on adobe bricks.

In this study, differently from [9], where the focus is modelling bi-modulus material beam subjected to three-point bending tests to study earth material behaviour, the problem at hand includes for the first time also the four-point layout assuming Euler–Bernoulli and Timoshenko beam models. Furthermore, in this study, an extensive experimental campaign for the mechanical characterisation of a lime mortar, including uni-axial tests, both in tension and in compression, as well as bending tests, provides significant data to validate the analytical model.

The adopted model considers the beam as constituted by two layers with different stiffnesses, separated from the neutral plane, which is shifted with respect to that of the mono-modulus beam. The integration of the differential problem, with the further unknown defined by the neutral plane position, leads to the closed-form solution of the displacement field, not yet available in the literature for the four-point layout.

The paper is organised as follows. In Section 2, assuming a bi-modulus material, a beam subjected to three-point and four-point concentrated loading is analysed to provide the complete displacement field. In Section 3, the experimental campaign is reported. The mechanical characterisation encompasses uni-axial compression and tension tests, three- and four-point bending tests, and on notched specimens three-point tests for mode-I fracture energy. For the interpretation of a series of four-point bending tests, deflection control has been carried out employing traditional contact measurement systems, i.e., displacement transducers and extensometers, and exploiting Digital Image Correlation (DIC) techniques. Discussion on test results is at the end of the section. Validation of the bi-modulus model for the interpretation of all the bending tests can be found in the following Section 4. The conclusions are at the end of the paper.

## 2. Bi-Modulus Beam under Three and Four-Point Bending

### 2.1. Statement and Solution of the Equilibrium Problem

In this section, the solution, in terms of displacements, for a pin-pin beam under one and two point-forces is evaluated. The beam’s material is assumed isotropic with different longitudinal elasticity modulus in compression, $E_{c}$, and tension, $E_{t}$. The tension to compression moduli ratio is expressed by the coefficient $n=E_{t}/E_{c}$. Accordingly, shear moduli and Poisson’s ratio are also different, $G_{c}=E_{c}/2\left(1+\nu_{c}\right)$ and $G_{t}=E_{t}/2\left(1+\nu_{t}\right)$. Provided the symmetry of the compliance stress tensor: $\nu_{t}/E_{t}=\nu_{c}/E_{c}$, it follows that $n=\nu_{t}/\nu_{c}$.

Starting from the displacement field associated with the models of Euler–Bernoulli (EBM) and Timoshenko (TM) beam, strain–displacement compatibility conditions and constitutive relations of the bi-modulus material are recalled to define the differential equilibrium problem. Differently from previous investigations, here, the solution in terms of displacement equations is retrieved for the pin-pin beam loaded by one and two point-forces.

**Figure 1:**
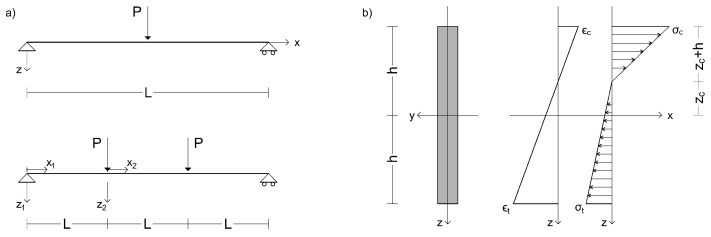
Scheme of the beam under three and four-point bending assuming a bi-modulus material ($E_{c}>E_{t}$): (**a**) reference systems, dimensions and load cases, (**b**) beam cross-section showing asymmetrical strains and stress through the height.

For the reference system shown in Figure 1, EBM includes displacement components in x- and z-directions, U(x,z) and W(x,z) respectively. For TM, the displacements components in x- and z-directions, $\tilde{U}\left(x,z\right)$ and $\tilde{W}\left(x,z\right)$, and rotation of the cross-section, $\tilde{\psi }\left(x\right)$, are included:

(1) $$\begin{array}{c} \begin{array}{cc} U(x,z) & =u\left(x\right)+z\;w^{\prime }\left(x\right)\\ W(x,z) & =w(x)\\ \tilde{U}\left(x,z\right) & =\tilde{u}\left(x\right)+z\;\tilde{\psi }\left(x\right)\\ \tilde{W}\left(x,z\right) & =\tilde{w}\left(x\right) \end{array} \end{array}$$

where *u* and *w*, are the x- and z-direction displacements, respectively, of the mid-line axis; superscript $\tilde{\cdot }$ is employed for displacement functions and any other variable denotes reference to TM and superscript ${\left(\right)}^{\prime }$ denotes x-derivative. Strain–displacement equations follow directly:

(2) $$\begin{array}{c} \begin{array}{ccc} \varepsilon_{x}\left(x,z\right) & =u^{\prime }\left(x\right)+z\;w^{\prime \prime }\left(x\right) & =\varepsilon_{0}\left(x\right)+z\;\varepsilon_{1}\left(x\right)\\ \gamma_{xz}\left(x,z\right) & =0 & \\ {\tilde{\varepsilon }}_{x}\left(x,z\right) & ={\tilde{u}}^{\prime }\left(x\right)+z\;{\tilde{\psi }}^{\prime }\left(x\right) & ={\tilde{\varepsilon }}_{0}\left(x\right)+z\;{\tilde{\epsilon }}_{1}\left(x\right)\\ {\tilde{\gamma }}_{xz}\left(x,z\right) & ={\tilde{w}}^{\prime }\left(x\right)+\tilde{\psi }\left(x\right) & ={\tilde{\gamma }}_{0}\left(x\right) \end{array} \end{array}$$

where $\varepsilon_{0}$ or ${\tilde{\varepsilon }}_{0}$, $\varepsilon_{1}$ or ${\tilde{\varepsilon }}_{1}$, and ${\tilde{\gamma }}_{0}$ are strains due to stretching, bending and shear, respectively. In this framework [27], the stress resultant and stress moments can be expressed, for the EBM and the TM, respectively, as:

(3) $$\begin{array}{c} \begin{array}{cc} N(x)= & \int_{-h}^{h}\sigma_{x}\left(x,z\right)\;dz=A\;\varepsilon_{0}\left(x\right)+B\;\varepsilon_{1}\left(x\right)\\ M(x)= & \int_{-h}^{h}\sigma_{x}\left(x,z\right)\;z\;dz=B\;\varepsilon_{0}\left(x\right)+D\;\varepsilon_{1}\left(x\right)\\ Q(x)= & -M^{\prime }\left(x\right)\\ \tilde{N}\left(x\right)= & \int_{-h}^{h}{\tilde{\sigma }}_{x}\left(x,z\right)\;dz=A\;{\tilde{\varepsilon }}_{0}\left(x\right)+B\;{\tilde{\varepsilon }}_{1}\left(x\right)\\ \tilde{M}\left(x\right)= & \int_{-h}^{h}{\tilde{\sigma }}_{x}\left(x,z\right)\;z\;dz=B\;{\tilde{\varepsilon }}_{0}\left(x\right)+D\;{\tilde{\varepsilon }}_{1}\left(x\right)\\ \tilde{Q}\left(x\right)= & \int_{-h}^{h}{\tilde{\tau }}_{xz}\left(x,z\right)\;dz=S\;\tilde{\gamma_{0}}\left(x\right) \end{array} \end{array}$$

where *A*, *B*, *D* and *S* are extensional, flexural–extensional coupling, flexural, and shear stiffnesses, respectively, defined as:

(4) $$\begin{array}{c} \begin{array}{cc} A= & \int_{-h}^{z_{c}}E_{c}\;dz+\int_{z_{c}}^{h}E_{t}\;dz=E_{c}\left(h+z_{c}\right)+E_{t}\left(h-z_{c}\right)\\ B= & \int_{-h}^{z_{c}}E_{c}zdz+\int_{z_{c}}^{h}E_{t}\;z\;dz=-\frac{1}{2}\left(E_{c}-E_{t}\right)\left(h^{2}-z_{c}^{2}\right)\\ D= & \int_{-h}^{z_{c}}E_{c}\;z^{2}\;dz+\int_{z_{c}}^{h}E_{t}\;z^{2}\;dz=\frac{1}{3}\left(E_{c}\left(h^{3}+z_{c}^{3}\right)+E_{t}\left(h^{3}-z_{c}^{3}\right)\right)\\ S= & K^{2}\int_{-h}^{z_{c}}G_{c}\;dz+\int_{z_{c}}^{h}G_{t}\;dz=\frac{5}{6}\left(G_{c}\left(h+z_{c}\right)+G_{t}\left(h-z_{c}\right)\right) \end{array} \end{array}$$

where $K^{2}$ is the shear correction factor, having assumed a compact cross-section, $z_{c}$, or $\tilde{z_{c}}$ in its place if the TM is referred, is the oriented distance of neutral axis, i.e., evaluated from z=0 towards the upper part of the cross-section, see Figure 1b, where the case of $z_{c}<0$ and compressed upper part are shown.

Then, employing Equation (3) in view of Equation (2), yields the differential equations of equilibrium:

(5) $$\begin{array}{c} \begin{array}{cc} N^{\prime }\left(x\right) & =A\;u^{\prime \prime }\left(x\right)+B\;w^{\prime \prime \prime }\left(x\right)=0\\ M^{\prime \prime }\left(x\right) & =B\;u^{\prime \prime \prime }\left(x\right)+D\;w^{iv}\left(x\right)=0\\ {\tilde{N}}^{\prime }\left(x\right) & =A\;{\tilde{u}}^{\prime \prime }\left(x\right)+B\;{\tilde{\psi }}^{\prime \prime }\left(x\right)=0\\ {\tilde{M}}^{\prime }\left(x\right)-\tilde{Q}\left(x\right) & =B\;{\tilde{u}}^{\prime \prime }\left(x\right)+D\;{\tilde{\psi }}^{\prime \prime }\left(x\right)-S\left({\tilde{w}}^{\prime }\left(x\right)+\tilde{\psi }\left(x\right)\right)=0\\ {\tilde{Q}}^{\prime }\left(x\right) & =S({\tilde{w}}^{\prime \prime }\left(x\right)+{\tilde{\psi }}^{\prime }\left(x\right))=0 \end{array} \end{array}$$

### 2.2. Three-Point Loading

Exploiting symmetry of loading and geometry, for the three-point bending condition of the EBM beam, to solve the system of Equation (5), the following seven boundary conditions (BC) must be satisfied:

(6) $$\begin{array}{c} \begin{array}{cc} w(0) & =u\left(L/2\right)=w^{\prime }\left(L/2\right)=0\\ N(L/2) & =M(0)=0\\ Q(0) & =P/2\\ M(L/2) & =PL/4 \end{array} \end{array}$$

For the TM beam, to solve the system of Equation (5), the following six boundary conditions (BC) must be satisfied:

(7) $$\begin{array}{c} \begin{array}{cc} \tilde{w}\left(0\right) & =\tilde{u}\left(L/2\right)=\tilde{\psi }\left(L/2\right)=0\\ \tilde{N}\left(L/2\right) & =\tilde{M}\left(0\right)=0\\ \tilde{Q}\left(0\right) & =P/2 \end{array} \end{array}$$

The systems of differential equations and related boundary conditions provide, for the EBM and the TM, respectively, the following displacement functions:

(8) $$\begin{array}{cc} U(x,z) & =\frac{P\left(L^{2}-4x^{2}\right)\left(Az-B\right)}{16\left(B^{2}-AD\right)} \end{array}$$

(9) $$\begin{array}{cc} W(x) & =\frac{AP(3L^{2}-4x^{2})x}{48(B^{2}-AD)} \end{array}$$

(10) $$\begin{array}{cc} \tilde{U}\left(x,z\right) & =\frac{P\left(L^{2}-4x^{2}\right)\left(Az-B\right)}{16\left(B^{2}-AD\right)} \end{array}$$

(11) $$\begin{array}{cc} \tilde{W}\left(x\right) & =\frac{AP(3L^{2}-4x^{2})x}{48(B^{2}-AD)}-\frac{Px}{2S} \end{array}$$

Displacements in the direction of the longitudinal axis, i.e, U(x,z) and $\tilde{U}\left(x,z\right)$ Equations (8) and (10), respectively, must be the same. Transverse displacements, i.e., W(x) and $\tilde{W}\left(x\right)$, Equations (9) and (11), respectively, differ for the part related to coefficient *S*. Neutral axis depth can be retrieved imposing that, for $z=z_{c}$ or $z={\tilde{z}}_{c}$, longitudinal strains, Equation (2) for the EBM and TM respectively, are zero. This yields, for both EBM and TM beams:

(12) $$z_{c}=h\left(1-\frac{2}{1+\sqrt{n}}\right)$$

in agreement with [16,29,31].

### 2.3. Four-Point Loading

For the four-point bending layout and exploiting the symmetry of loading and geometry, the piece-wise continuity of stress resultant and stress moments is encountered, Figure 1. Therefore, the system of ODE (Equation (5)), must be defined on the first (i.e., $u_{1}\left(x\right)$, $w_{1}\left(x\right)$, $\tilde{u_{1}}\left(x\right)$, $\tilde{w_{1}}\left(x\right)$, $\tilde{\psi_{1}}\left(x\right)$) and on the second branch ($u_{2}\left(x\right)$, $w_{2}\left(x\right)$, $\tilde{u_{2}}\left(x\right)$, $\tilde{w_{2}}\left(x\right)$, $\tilde{\psi_{2}}\left(x\right)$). Boundary conditions must comply with constraints disposition, stress resultant values and continuity conditions on displacements among the two branches. For the EBM and TM, respectively, boundary conditions of the four-point bending layout are:

(13) $$\begin{array}{c} \begin{array}{cc} w_{1}\left(0\right) & =u_{2}\left(L/2\right)=w_{2}^{\prime }\left(L/2\right)=0\\ Q_{2}\left(L/2\right) & =M_{1}\left(0\right)=N_{1}\left(L\right)=N_{2}\left(L/2\right)=0\\ Q_{1}\left(0\right) & =P\\ M_{1}\left(L\right) & =M_{2}\left(0\right)=M_{2}\left(L/2\right)=PL\\ u_{1}\left(L\right) & =u_{2}\left(0\right)\\ w_{1}\left(L\right) & =w_{2}\left(0\right)\\ w_{1}^{\prime }\left(L\right) & =w_{2}^{\prime }\left(0\right)\\ \tilde{w_{1}}\left(0\right) & =\tilde{u_{2}}\left(L/2\right)=\tilde{\psi_{2}}\left(L/2\right)=0\\ \tilde{N_{1}}\left(L\right) & =\tilde{N_{2}}\left(L/2\right)=0\\ \tilde{Q_{1}}\left(0\right) & =P\\ \tilde{Q_{2}}\left(L/2\right) & =\tilde{M_{1}}\left(0\right)=0\\ \tilde{M_{2}}\left(L/2\right) & =PL\\ \tilde{u_{1}}\left(L\right) & =\tilde{u_{2}}\left(0\right)\\ \tilde{w_{1}}\left(L\right) & =\tilde{w_{2}}\left(0\right)\\ \tilde{\psi_{1}}\left(L\right) & =\tilde{\psi_{2}}\left(0\right) \end{array} \end{array}$$

Employing Equations (5) and (13) on the corresponding sub-domains (i.e., $x_{1}$ and $x_{2}$) provides the following set of displacement fields for the EBM and TM, respectively:

(14) $$\begin{array}{cc} U_{1}\left(x,z\right) & =\frac{P\left(2x^{2}-L^{2}\right)\left(B-Az\right)}{2(B^{2}-AD)} \end{array}$$

(15) $$\begin{array}{cc} W_{1}\left(x\right) & =\frac{APx(x^{2}-6L^{2})}{6(B^{2}-AD)} \end{array}$$

(16) $$\begin{array}{cc} U_{2}\left(x,z\right) & =\frac{LP(2x-L)(B-Az)}{2(B^{2}-AD)} \end{array}$$

(17) $$\begin{array}{cc} W_{2}\left(x\right) & =\frac{ALP(3x^{2}-3Lx-5L^{2})}{6(B^{2}-AD)} \end{array}$$

(18) $$\begin{array}{cc} \tilde{U_{1}}\left(x,z\right) & =\frac{P\left(2x^{2}-L^{2}\right)\left(B-Az\right)}{2(B^{2}-AD)} \end{array}$$

(19) $$\begin{array}{cc} \tilde{W_{1}}\left(x\right) & =\frac{APx(x^{2}-6L^{2})}{6(B^{2}-AD)}+\frac{Px}{S} \end{array}$$

(20) $$\begin{array}{cc} \tilde{U_{2}}\left(x,z\right) & =\frac{LP(2x-L)(B-Az)}{2(B^{2}-AD)} \end{array}$$

(21) $$\begin{array}{cc} \tilde{W_{2}}\left(x\right) & =\frac{ALP(3x^{2}-3Lx-5L^{2})}{6(B^{2}-AD)}+\frac{PL}{S} \end{array}$$

Neutral axis depth has the same expression of Equation (12).

### 2.4. Sensitivity Analysis for Variation of Coefficient *n*

**Figure 2:**
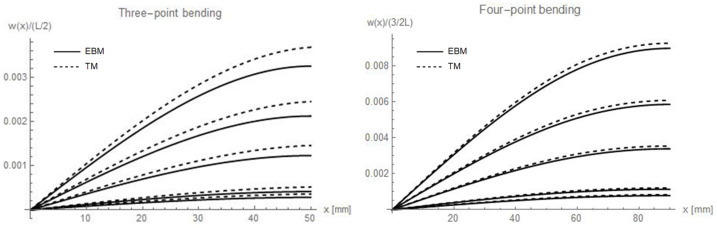
Normalised deflections with respect to the half of the total free length of the beams, i.e., L/2 = 50 mm for three-point bending and 3/2 L = 90 mm for four-point bending, along *x* axis, varying values of *n*. The fixed value of the load is 2000 N, while the assumed *n* values are, from top to bottom, respectively *n* = 0.03; 0.05; 0.1; 0.5 and 1 (which is the mono-modulus beam).

In order to highlight the effect of the variation of coefficient *n* on the vertical displacements of beams, Figure 2 reports the displacements normalised with respect to the half of the total free length, i.e., L/2 for three-point bending and 3/2 L for four-point bending (as depicted in Figure 1), along the *x* axis. Since $E_{c}$ > $E_{t}$, in the admissibility domain for n, 0 < *n* < 1, the cases *n* = 0.03; 0.05; 0.1; 0.5 were assumed, in addition to *n* = 1, the mono-modulus beam, for comparison.

The diagrams of Figure 2 allow observing that the lower the *n* value, the higher the estimated displacement. Moreover, the difference between the Euler–Bernoulli and the Timoshenko models, for which higher deflections are obtained, increases as the *n* decreases. This is especially true for the three-point bending, which is more affected by the shear effect.

## 3. Materials and Methods

### 3.1. Specimens and Tests

The commercially available premixed dry mortar, Kerakoll GeoCalce^®^F (class M15 according to [32]) employed in the experimental campaign contains pure NHL 3.5 certified natural lime, natural river-washed fine (0.1–0.5 mm) and medium-grained (0.1–1 mm) siliceous sand, dolomitic limestone (0–1.4 mm), white Carrara marble (0–0.2 mm) and mineral geo-binder. The water-to-mortar ratio used is 1:5.5, indications provided in the data sheet [33], were followed during slurry preparation. The following samples were considered in the experimental campaign:

- SP—short prisms (dim. 40 × 40 × 160 mm${}^{3}$), 9 specimens, using standard moulds
- LP—long prisms (dim. 40 × 40 × 240 mm${}^{3}$), 9 specimens, using specifically fabricated plexiglass moulds
- C—cubic prisms (dim. 40 × 40 × 40 mm${}^{3}$), 11 specimens, using standard moulds
- SPN—short prisms with a notch (dim. 40 × 40 × 160 mm${}^{3}$), 3 specimens, using standard moulds and a specifically designed device to create, on the middle of three of the longest faces, a 3 mm thick and 10 mm high notch with a sharp edge, which ensures the trigger for a stable vertical crack plane and avoids possible damages to the specimens connected to cutting operations after the curing phase.

After the specimens had been removed from the moulds they were left drying for at least 28 days in a controlled environment (20${}^{\circ }$ and 60% R.H.). All the tests were carried out employing an Instron Satec (InS) with 600 KN capacity or a Zwick Roell-Z100 (ZR) with 100 KN capacity test press employing a parallel Keniuko compression load cell with 20 KN (C2) capacity or a Keniuko Tension load cell with 5 KN (T5) capacity; the tests carried out and the related setups are:

- TM—Uniaxial tensile test for the determination of Young’s modulus in tension on part of the LP sample (3 specimens) carried out on the ZR-T5 test press and assigning monotonic loading with a speed of $3\times 10^{-4}$ MPa/s up to 160 N (pre-load phase) and then with a constant monotonic speed of 0.01 MPa/s up to the rupture of the specimen. Specimens were fixed to the doubly hinged test apparatus employing the Sikadur 31CF bi-component epoxy adhesive. Specimens were instrumented with two CE-DT on the head of the tightening apparatus, one 50 mm and two 100 mm omega extensometers which were removed before the end of the test.
- CM—Uniaxial compression test for the determination of Young’s modulus in compression on part of the SP sample (6 specimens) carried out on the ZR-C2 press according to Method 2 of [34]; in particular, for the three pre-loading cycles 0.5 MPa and 5 MPa were set as stress bounds at 0.4 MPa/s (which corresponds to 640 N/s), the same speed was kept for the loading up rupture. Two Cantilever Displacement Transducers (CE-DT) were placed on the upper loading plate separated from the test press head by a steel sphere. To record strains in the central part of the specimen two 50 mm omega extensometers were installed, and removed before the end of the tests.
- CS—Uniaxial compression test for the determination of the compressive strength (6 specimens obtained from 3PB-SP specimens stumps), according to [1] employing a 50 N/s load speed on the InS test press
- 3PB—Three-point bending test for the determination of deflection on part of the SP sample (3 specimens) carried out according to [1] on the ZR-C2 test press choosing a load application speed of 40 N/s. Specimens were instrumented with two Cantilever Displacement Transducers (CE-DT) at the loading point.
- IF—Mode-I Fracture energy three-point bending test on the SPN sample carried out on the ZR-C2 test press at a load speed of $5\times 10^{-5}$ mm/s. Specimens were instrumented with two Cantilever Displacement Transducers (CE-DT) at the loading point free to rotate through a steel sphere, and two clip gauges inserted in the notch to monitor the Crack Mouth Opening Displacement (CMOD).
- 4PB—Four-point bending test for the determination of deflection on part of the LP sample (3 specimens) carried out on InS test press setting a constant spacing among steel cylinders equal to 60 mm and leaving 30 mm free from the edges. Tests were carried out in displacement control with 40 N/s speed. Specimens were instrumented with two CE-DT placed on the steel plate transferring the load to the upper cylinders.
- 4PB-DIC—Four-point bending test using Digital Image Correlation for the determination of deflection on part of the LP sample (3 specimens) carried out on the ZR-C2 test press, the same test layout of 4PB (60 mm spacing and 30 mm from the edges) was considered. Tests were carried out in displacement control with 4 N/s speed to enable to shoot a sufficient number of images during the tests. The speckled area is the central part of the specimen (80 × 40 mm${}^{2}$), circular speckles were created randomly and did not exceed 0.05 mm in diameter. Images were acquired every 20 s through a Canon EOS550D and a SIGMA DC 17–70 mm placed at 500 mm distance from the specimen, focused at 44 mm with f/9 exposure for 1/6 s. The specimen was enlightened with a 4000-lumen halogen bulb.

**Figure 3:**
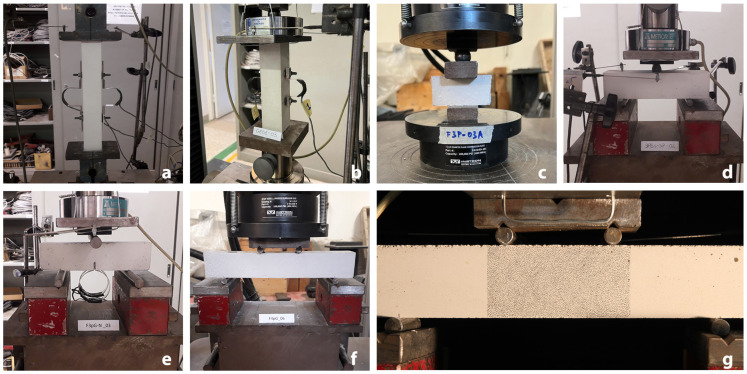
Test set ups: (**a**) uniaxial tensile for the determination of Young’s modulus in tension, (**b**) compression for the determination of Young’s modulus in compression, (**c**) compression for the determination of compressive strength, (**d**) three-point bending, (**e**) three-point bending with a notched specimen, (**f**) four-point bending, (**g**) four-point bending with measurement DIC system.

For each type of test, sources of uncertainty were identified. In particular, with regard to specimens, a source of uncertainty concerns the use of a calliper with a tolerance of 0.02 mm for measuring the dimensions; for the test system the sources consist of the alignment system, force measurement accuracy (cell sensitivity 2 mV/V), and extensometer accuracy (omega-shaped extensometer 2130 × 10${}^{-6}$/mm in tension and 2150 × 10${}^{-6}$/mm in compression; cantilever displacement transducers 592 × 10${}^{-6}$/mm, clip gauges 676 × 10${}^{-6}$/mm); as for the environment, the ambient temperature and the humidity are considered; finally with regard to test procedure, in addition, to load balancing (zeroing), the speed of load application was considered (Figure 3).

### 3.2. Test Results and Discussion

#### 3.2.1. TM-LP

**Figure 4:**
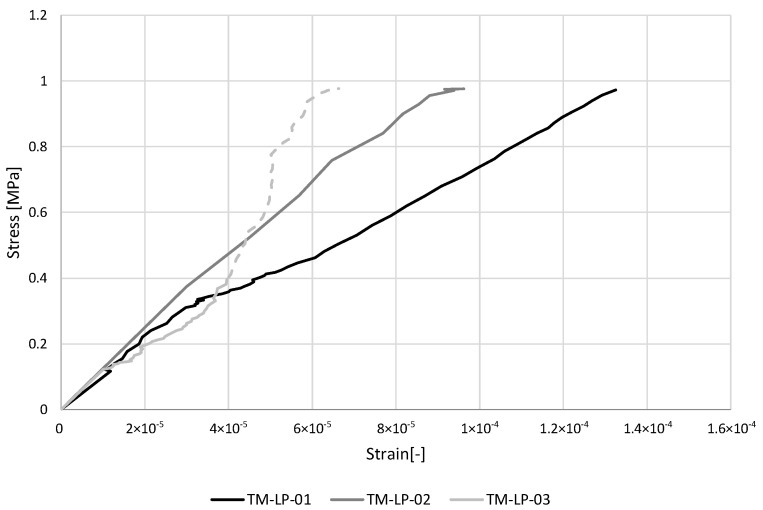
Stress–strain diagrams for uniaxial tensile tests (TM-LP). The last part of the diagram of specimen TM- LP-03 is dashed due to a recording error, and it has not been considered for the evaluation of the elasticity modulus.

The stress–strain diagrams recorded by omega extensometers, hence, up to 1 MPa, and the results of uniaxial tensile tests are reported respectively in Figure 4 and Table 1. The value of tension Young’s modulus of each specimen was evaluated as the average of the values obtained by the three omega extensometers used to instrument the samples, as the tangent modulus between the lower and upper-stress thresholds. The mean value is $E_{t}$ = 7422 MPa with a CV of 21%. The average peak load is P = 3557 N with a CV of 11%. The stress reached at the peak load is on average $\sigma_{t}$ = 2.22 MPa.

#### 3.2.2. CM-SP

**Figure 5:**
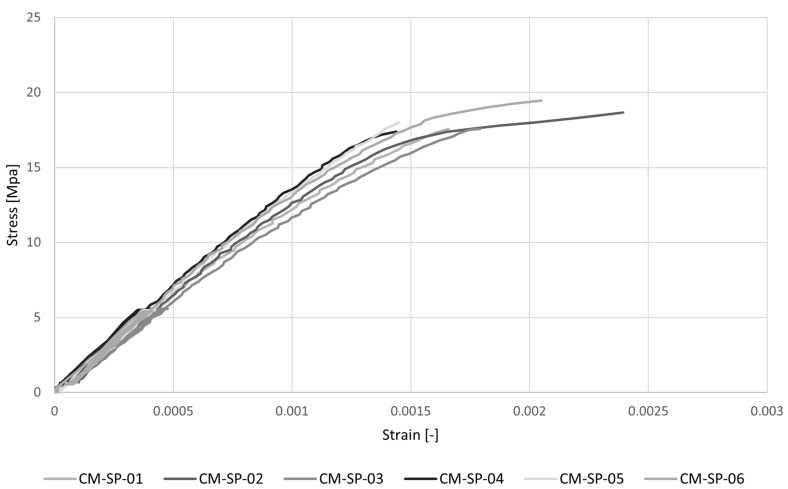
Stress–strain diagrams for uniaxial compression tests (CM-SP) for the determination of Young’s modulus in compression.

The stress–strain diagrams recorded by omega extensometers and the results of uniaxial compression tests for the determination of Young’s modulus are reported respectively in Figure 5 and Table 2. The average value of the modulus, evaluated, as the tangent modulus between the lower and upper-stress thresholds, according to [34], is $E_{c}$ = 14,384 MPa with a low variation (CV = 7%). The mean value of the peak stress is $\sigma_{c}$ = 18.66 MPa with a CV = 7%.

#### 3.2.3. CS-C

**Figure 6:**
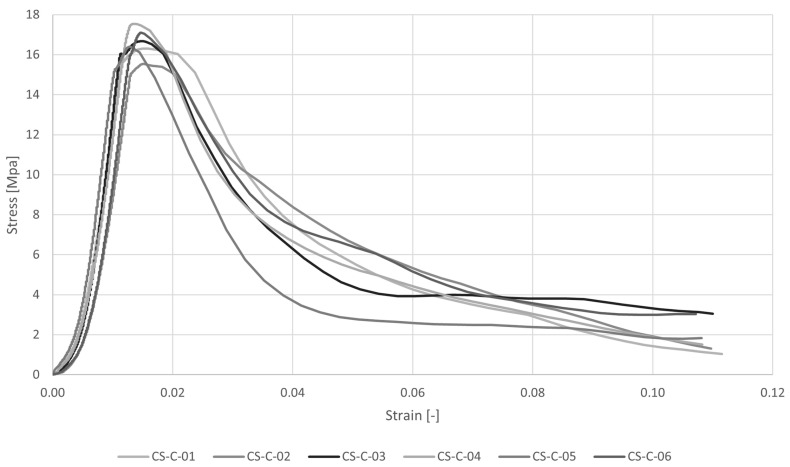
Stress–strain diagrams for uniaxial compression tests (CS-C).

The stress–strain diagram and the results of uniaxial compression tests are reported respectively in Figure 6 and Table 3. The average peak load is P = 26,554 N and the maximum compression strength is $\sigma_{c}$ = 16.60 MPa with very low variation, CV = 4%.

Although stocky specimens reach greater strength compared to more slender ones, the different speed load applied to the CS-C (50 N/s) and CM-SP (640 N/s) tests has to lead to lower values, about 10%, for CS-C compared to CM-SP. From experimental results, as reported in [35], the compressive strength increases as the load rate increases. This could be explained by the fact that, at high-speed loading, cracks propagate along greater resistance paths because they do not have sufficient time to search the path of minimum resistance [35].

#### 3.2.4. 3PB-SP

**Figure 7:**
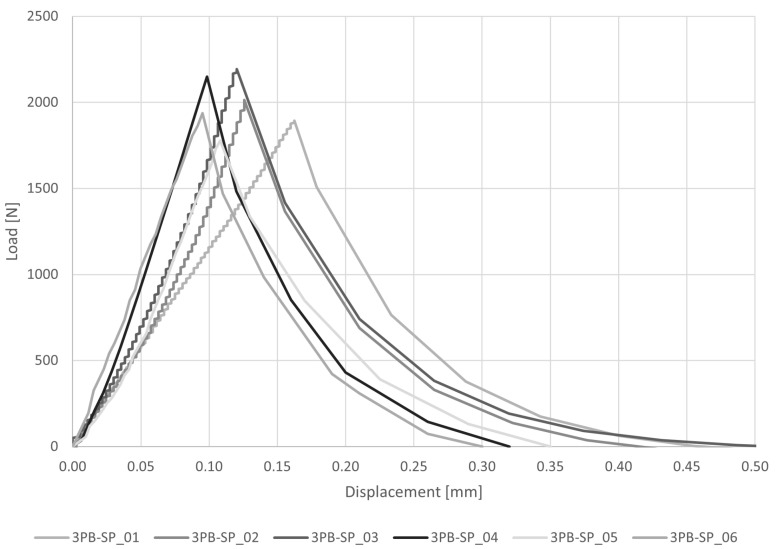
Load–displacement diagrams for three-point bending tests (3PB-SP).

The load–displacement diagram and the results of three-point bending tests are reported respectively in Figure 7 and Table 4. The average peak load is P = 1994 N and the associated average peak stress is $\sigma_{f}$ = 4.67 MPa, with a CV = 8%. The flexural stress here reported was evaluated using Navier’s formulation. The average vertical displacement under the loading point is η = 0.1068 mm with a CV = 26%. The high value of the coefficient of variation is due to the fact that the deflection under the loading point of specimen 3PB-SP_01 is almost two times the deflections of the others.

#### 3.2.5. IF-SPN

**Figure 8:**
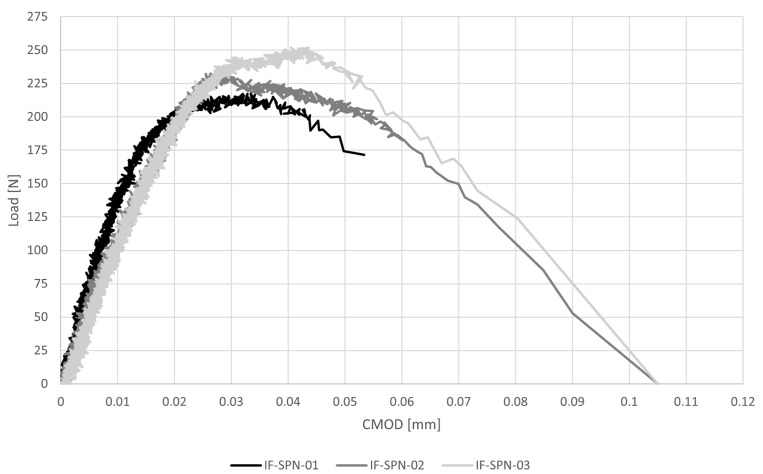
Load-crack mouth opening displacement response for mode- I fracture energy three-point bending tests (IF-SPN).

The load-crack mouth opening displacement diagram and the results of mode-I fracture energy three-point bending tests are reported respectively in Figure 8 and Table 5. The peak load is P = 234 N and the average peak stress is $\sigma_{f}$ = 1.17 MPa, with a CV = 7%. The load is much lower than the one obtained in the 3PB-SP tests, due to the presence of the notch that makes the SPN samples more slender than the SP and forces specimens to fracture at the edge. Moreover, the lower loading rate employed, more than two orders of magnitude lower than that employed for the 3PB-SP, also affect the peak load recorded, as reported, e.g., in [35,36]. The average value of the fracture energy, calculated as the area under the stress versus crack opening displacement curve from peak stress up to displacement corresponding to 1% of the peak stress, is $G_{F}$ = 7.50 N/m with a CV of 24%. This value is coherent with the experimental results obtained on hydraulic mortars in [37,38].

#### 3.2.6. 4PB-LP

**Figure 9:**
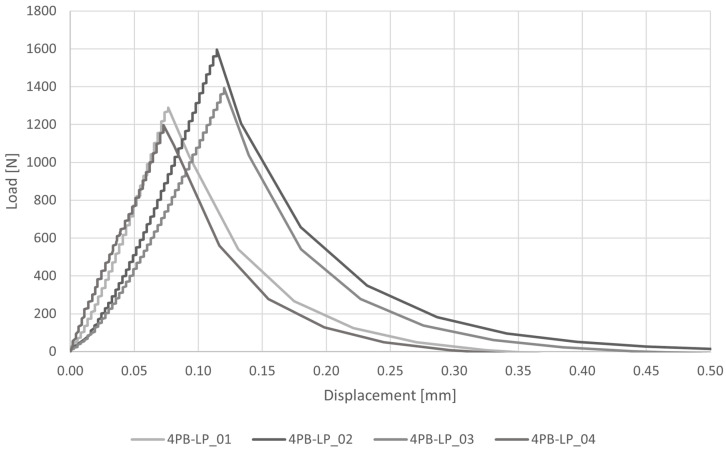
Load–displacement diagram for four-point bending tests (4PB-LP).

The load–displacement diagram and the results of four-point bending tests are reported respectively in Figure 9 and Table 6. The average peak load is P = 1369 N and the average peak stress is $\sigma_{f}$ = 3.85 MPa, with a CV = 12%. The flexural stress here reported was evaluated using Navier’s formulation. The average deflection at the loading points is η = 0.0820 mm with a CV = 20%.

#### 3.2.7. 4PB-DIC-LP

**Figure 10:**
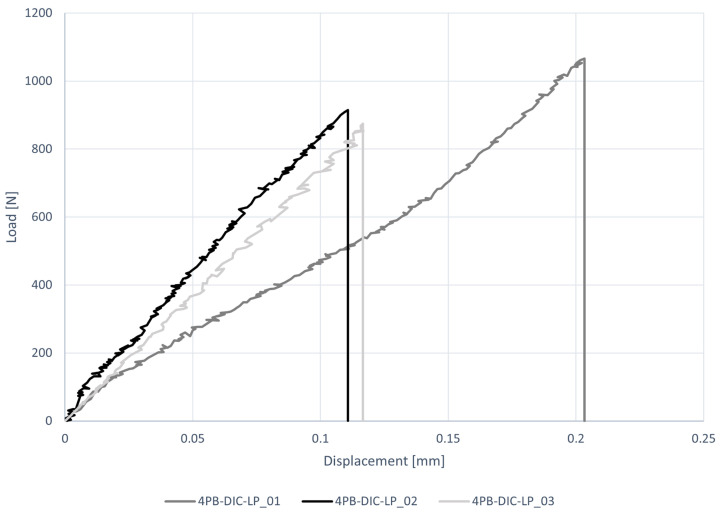
Load–displacement diagrams for four-point bending tests (4PB-DIC-LP).

The load–displacement diagram and the results of four-point bending tests carried out exploiting contactless measurement systems (DIC), are reported respectively in Figure 10 and Table 7. The average peak load is P = 952 N and the average peak stress is $\sigma_{f}$ = 2.68 MPa, with a CV = 11%. The reported flexural stress was evaluated using Navier’s formulation. The deflection values of each specimen reported in Table 7 are the average of the vertical displacements recorded under the two loading points. In particular, the average values of $\eta^{DIC}$ were taken as the mean of those recorded in a 4.5 mm square area under the cylinders. CE-DT and DIC provide different mean values with different CV: $\eta^{CE}$ = 0.1435 mm with a CV of 36% and $\eta^{DIC}$ = 0.1240 mm with a CV of 15%. In Table 7 it can be seen that the deflections under the two loading points of the specimen 4PB-DIC-LP_02 and 4PB-LP-DIC_03 obtained from DIC and CE-DT are similar (CV = 0.1–0.5%), while specimens of the first sample are more distant with a CV of 23%. The higher value read by transducers can be explained by the fact that they were placed on the steel plate that transfers the load to the upper cylinders, therefore some additional displacement could be read.

**Figure 11:**
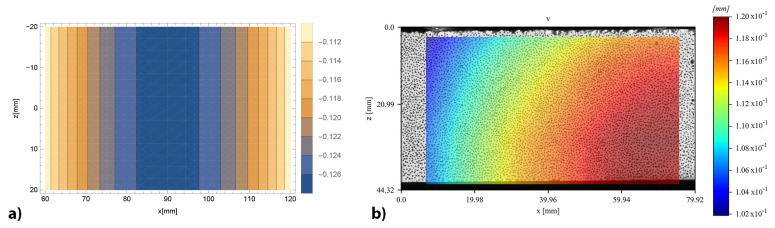
Vertical displacements contour of 4PB-DIC-LP_02 sample: (**a**) prevision obtained by the bi-modulus model (**b**) results obtained by DIC analysis.

**Figure 12:**
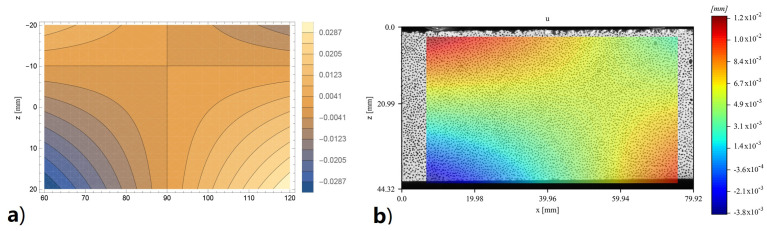
Contour of horizontal displacements of 4PB-DIC-LP_02 sample: (**a**) prevision obtained by the bi-modulus model (**b**) results obtained by DIC analysis.

The complete displacement fields, both vertical and horizontal, provided by DIC and obtained by the bi-modulus model through the displacement functions (Equations (14)–(21)), are reported for the specimen 4PB-DIC-LP_02 respectively in Figure 11b and Figure 12b. As can be seen in Figure 11b, the sample has shown a 15% higher deflection under the right cylinder. For this reason, the bands of colour, representing the same vertical displacement values, are not vertical nor specular to the centre of the specimen, as supposed to be (Figure 11a). The difference between the deflections under the two loading points also leads to a visible shifting of the vertical symmetry axis in the horizontal displacements (Figure 12b), which usually corresponds to the middle of the specimen (Figure 12a), towards the right. Nevertheless, the displacement distribution along the surface is clearly visible: since the upper part is compressed, there is a movement towards the centre of the specimen, while in the lower one, which is in tension, there is a movement towards the sides.

The 4PB-DIC-LP tests have reached a lower peak load (−28%) but higher deflection (+92% read by transducers and +56% read by DIC) than 4PB-LP, which were carried out with a speed 10 times faster. As reported in [39], from experimental results, the peak load decreases as the loading rate decreases, while the displacement increases.

## 4. Validation of the Analytical Model to the Experimental Results

### 4.1. Overview of Model Validation

The analytical bi-modulus models described in Section 2 are applied to the beam experimental specimens subjected to bending tests. In particular, assuming X-X-YY as notation, with X-X the sample specification and YY the beam model specification, EB for Euler–Bernoulli and TM for Timoshenko, the following evaluations were carried out on:

- four beam models of three-point bending tests:
  - 3PB-SP-EB and 3PB-SP-TM, with free span-to-height ratio 2.5;
  - IF-SPN-EB and IF-SPN-TM with free span-to-height ratio 3.3.
- two beam models of four-point bending tests, 4PB-LP-EB and 4PB-LP-TM with the ratio free span–height 4.5.

The elastic modulus in tension and compression determined experimentally with the uniaxial tests, TM and CM, respectively, were employed in order to provide $n=E_{t}/E_{c}$. For each beam model, Table 8 shows estimations of the maximum values of the tensile and compressive stress, the position of the neutral axis and the vertical displacement of the external load application point, assuming the maximum average load obtained experimentally for 3PB-SP and 4PB-LP, and assuming the average load at the end of the linear phase (P = 157 N) for IF-SPN. The relative errors between estimations of the mono- (MM) or bi-modulus (EM, TM) models and the recorded experimental displacement are also reported to highlight the ability of analytical models to interpret the experimental behaviour in bending. In Table 8, the results relating to the mono-modulus beam model are also reported with the aim of comparison with the bi-modulus ones, using, for the displacement estimation, the experimental compressive modulus as usually assumed.

The analytical bi-modulus models described in Section 2 are applied to the beam experimental specimens subjected to bending test also in an indirect way to estimate the elastic modulus in tension, having assumed the modulus in compression, and by exploiting experimental load and displacement data (Table 9). The bi-modulus interpretation of the bending test could in this way provide an estimation of the elastic modulus in tension, allowing to avoid uniaxial tensile tests which, as known, in addition to anchoring problems to the test apparatus, exhibit difficulties in implementing testing procedures that provide sufficiently long-lasting uni-axial tensile stress. Therefore, for each bending test, the experimental values of displacement and load allow the calibration of the analytical model by the displacement functions (Equations (8)–(11) and (14)–(21)), assuming the compressive Young’s Modulus determined experimentally.

Moreover, inputting experimental tension and compression moduli into linear FEM environment provides consistent data with respect to uniaxial tests in tension and in compression, respectively.

### 4.2. Estimation of the Displacements and the Stress State

Table 8 collects the results of the analyses carried out on the analytical models, using $n=E_{t}/E_{c}=7422/14,384=0.52$, determined by uniaxial tests. Each analytical model was calibrated with the corresponding experimental average load P and provided displacements and stress state. The error in estimating the average experimental displacement, $\delta_{BM}$, was then calculated for each model as [($\eta_{exp}-\eta_{BM}$)/$\eta_{exp}$], where $\eta_{exp}$ is the experimental displacement and $\eta_{BM}$ is the bi-modulus displacement. That error has been also compared with the one obtained from the estimate of the displacement calculated with the classical mono-modulus beam model, $\delta_{MM}$, based on the unique compressive Young’s Modulus.

As regards the comparison between the experimental and analytical displacements, Table 8 shows that, in general, the TM models offer, as expected, a better estimate than EB models, considering also the shear deformability. Among the TM models, the 4PB-LP model produced a better estimate of the displacement with an error of 28%. On the contrary, the 3PB-SP-TM showed an error of 81%. The results of the IF-SPN-TM models provide an intermediate error (61%) between the other two models, suggesting a link between the results obtained and the free span–height ratio of the samples. As known, the beam behaviour implies a free span–height ratio greater than or equal to 4 and the samples 3PB-SP and IF-SPN have a geometric shape ratio of 2.5 and 3.3, respectively. For this reason, the experimental behaviour of 3PB-SP and IF-SPN deviated further from the ideal one, differently from the model 4PB-LP with a free span–height ratio of 4.4.

As regards the relative errors of the vertical displacements evaluated with the classical mono-modulus beam theory, $\delta_{MM}$, it is possible to observe, also in this case, that they increase with the decrease of the beam free span–height ratio. The errors, $\delta_{MM}$, are respectively 50% for 4PB-LP, 82% for IF-SPN and 87% for 3PB-SP.

As shown in Table 8, the bi-modulus models offer a better estimation of the vertical displacement than the mono-modulus theory. In fact, the bi-modulus relative errors, $\delta_{BM}$, are lower than the mono-modulus ones, $\delta_{MM}$. In particular, the Timoshenko models show values 7% lower for 3PB-SP, 44% lower for 4PB-LP and 26% lower for IF-SPN.

Concerning the stress state, the different stress values in tension and in compression induced by the different moduli ($E_{c}>E_{t}$) cause a shifting of the neutral axis towards the compressed area and a different distribution of stress, which becomes neither equal nor symmetric with respect to the neutral axis. Tensile stress estimated by means of the bi-modulus models shows lower values than their single-modulus counterparts. In particular, the mono-modulus model provides tensile stress 16% higher than the values obtained by the bi-modulus models, which compared to the strength value obtained in the uniaxial tensile tests are respectively: 110% higher for 3PB-SP model, 75% higher for 4PB-LP model and 65% lower for IF-SPN model. Instead, the bi-modulus models provide closer values to the tensile strength value obtained in the uniaxial tests. Indeed they are respectively 80% higher for 3PB-SP model, 49% higher for 4PB-LP model and 70% lower for IF-SPN model.

### 4.3. Estimation of the Elastic Modulus in Tension

Table 9 collects the results of the analyses applied to the three- and four-point bending tests using the indirect way, and the flexural elastic modulus $E_{f}$ evaluated by the classical mono-modulus beam model. Each analytical model was calibrated with the experimental average peak load P and the corresponding vertical displacement $\eta_{exp}$, assuming the experimental value of the modulus in compression $E_{c}$ = 14,384 MPa. In this way, the elastic modulus in tension can be determined. The error in estimating the experimental elastic modulus in tension ($E_{t_{exp}}$ = 7422 MPa) was calculated for each model as $\delta_{E_{t}}$ = [($E_{t_{exp}}-E_{t}$)/$E_{t_{exp}}$], where $E_{t}$ is the elastic modulus in tension analytically estimated.

**Figure 13:**
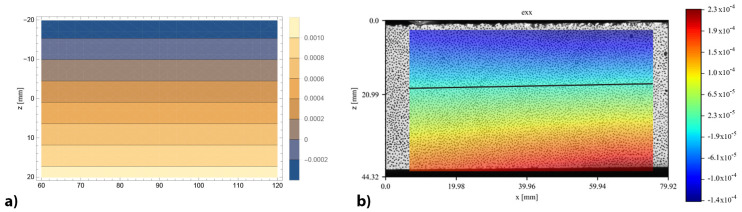
Longitudinal strain contour of 4PB-DIC-LP_02 sample: (**a**) prevision obtained by the bi-modulus model (**b**) results obtained by DIC analysis, the black line represents the position of the neutral axis.

The results related to the bi-modulus models for the estimation of the elastic modulus in tension confirm the trend of those obtained in the first application (Section 4.2): the best results are obtained with the TM models and the error decreases as the beam shape ratio increases. In particular, the closest value to the experimental result is that of the 4PB-LP-TM model, which is 32% lower than $E_{t_{exp}}$ = 7422 MPa. On the contrary, the 3PB-SP-TM model shows the highest error (88%), while the IF-SPN-TM model provides an intermediate value with an error of 74%. As can be seen in Table 9, the lower the elastic modulus in tension, the higher the shifting of the neutral axis towards the compressed area. The shifting of the neutral axis of approximately 4 mm obtained from the DIC analysis can be seen in Figure 13b, providing a depth of the stretched height of the section equal to 24 mm.

## 5. Conclusions

In this article, a bi-modulus beam model has been defined and implemented to provide a proper interpretation of three- and four-point bending tests on mortars. The model has been applied to standard three- and four-point bending tests, and mode-I fracture energy tests (three-point bending on notched specimens), all being part of the experimental campaign reported here and carried out on lime mortar. The experimental campaign, which also included uni-axial tension and compression tests, has enabled the evaluation of the mechanical characteristics of the material.

Assuming both Euler–Bernoulli and Timoshenko beam models, the proposed bi-modulus model considers the beam as constituted by two layers with different stiffnesses. Because of this assumption, the neutral plane between these layers is shifted with respect to that of the mono-modulus beam. The closed form of the displacement field is determined by the integration of the differential problem, with the further unknown defined by the neutral plane position. The closed form of the displacement field for the four-point loaded bi-modulus beam is presented here for the first time.

To interpret experimental bending test results by the bi-modulus model, two applications are carried out. In the first application, the coefficient *n* relating the tension to compression elasticity modulus was assumed exploiting the experimental values obtained with uniaxial tests. The estimated displacements were compared with those experimentally recorded in the bending tests. Comparisons were also made on the bi-modulus stress state with the mono-modulus one and the tensile strength obtained from the uniaxial test. In the second application, the experimental displacements of the bending tests were employed to calibrate the analytical models and obtain the estimate of the elastic modulus in tension, assuming the modulus in compression.

The difference between the elastic moduli, with $E_{c}>E_{t}$, as shown experimentally, induces a shift of the neutral axis towards the compressed area and an asymmetric distribution of the stresses with respect to the neutral axis. The shifting of the neutral axis has been directly observed through the DIC analysis of images taken during three four-point bending tests carried out at a loading rate enabling a sufficient number of pictures to be shot.

Compared to the tensile strength determined experimentally, the bi-modulus models estimate closer values than those evaluated with the standard formula. It is worth highlighting that the error between analytical and experimental results changes with the test considered. The bi-modulus interpretation of tests that provides results closer to the experimental values, both in terms of elastic moduli and strength values, is related to the four-point bending layout. In fact, the four-point bending specimen shows higher slenderness compared to the three-point layout. Furthermore, the effect of the concentrated load is attenuated along the central part of the specimen subject to the four-point layout, differently from what occurs in the three-point layout.

The comparison with the experimental results allows affirming that the bi-modulus interpretation of the bending test could allow the use of the bending test for the estimation of the elastic modulus in tension. In so doing, the uniaxial tensile tests could be avoided, simplifying experimentation since, as known, the tensile test shows difficulties in implementing testing procedures that provide sufficiently long-lasting uni-axial tensile stress and specimen anchoring to the test apparatus.

## Figures and Tables

**Table 1:** Results of uniaxial tensile tests (TM-LP) for the determination of Young’s Modulus in tension, $E_{t}$. P is the maximum force and $\sigma_{t}$ is the maximum tension stress.

	Et [MPa]	P [N]	$\sigma_{t}$ [MPa]
TM-LP_01	6341	3765	2.35
TM-LP_02	8772	3100	1.94
TM-LP_03	7692	3805	2.38
Av	7422	3557	2.22
CV	21%	11%	11%

**Table 2:** Results of uniaxial compression tests (CM-SP) for the determination of Young’s Modulus in compression, $E_{c}$. P is the maximum force and $\sigma_{c}$ is the maximum compressive stress.

	$E_{c}$ [MPa]	P [N]	$\sigma_{c}$ [MPa]
CM-SP_01	13,395	28,090	17.56
CM-SP_02	14,290	29,875	18.67
CM-SP_03	12,963	28,135	17.58
CM-SP_04	15,219	28,415	17.76
CM-SP_05	15,091	33,437	20.90
CM-SP_06	15,343	31,142	19.46
Av	14,384	29,849	18.66
CV	7%	7%	7%

**Table 3:** Results of uniaxial compression tests (CS-C) in terms of maximum load P and maximum compression stress $\sigma_{c}$.

	P [N]	$\sigma_{c}$ [MPa]
CS-C_01	26,096	16.31
CS-C_02	24,861	15.54
CS-C_03	26,678	16.67
CS-C_04	28,072	17.55
CS-C_05	26,251	16.41
CS-C_06	27,364	17.10
Av	26,554	16.60
CV	4%	4%

**Table 4:** Results of three-point bending tests (3PB-SP) in terms of maximum load P and corresponding vertical displacement η and flexural stress $\sigma_{f}$ according to the classical mono-modulus beam model.

	P [N]	$\sigma_{f}$ [MPa]	η [mm]
3PB-SP_01	1893	4.44	0.1626
3PB-SP_02	2014	4.72	0.0869
3PB-SP_03	2194	5.14	0.0900
3PB-SP_04	2148	5.03	0.0984
3PB-SP_05	1777	4.16	0.1080
3PB-SP_06	1937	4.54	0.0952
Av	1994	4.67	0.1068
CV	8%	8%	26%

**Table 5:** Results of mode-I fracture energy three-point bending tests (IF-SPN) in terms of maximum peak load P, corresponding flexural stress $\sigma_{f}$ according to the classical mono-modulus beam model, corresponding vertical displacement η, fracture energy $G_{F}$ and crack mouth opening displacement, CMOD.

	P [N]	$\sigma_{f}$ [MPa]	$G_{F}$ [N/m]	CMOD [mm]	η [mm]
IF-SPN_01	218	1.11	6.40	0.033	0.0215
IF-SPN_02	233	1.13	9.61	0.028	0.0258
IF-SPN_03	252	1.26	6.48	0.044	0.0348
Av	234	1.17	7.50	0.035	0.0274
CV	7%	7%	24%	22%	25%

**Table 6:** Results of four-point bending tests (4PB-LP) in terms of maximum load P and corresponding vertical displacement η and flexural stress $\sigma_{f}$ according to the classical mono-modulus beam model.

	P [N]	$\sigma_{f}$ [MPa]	η [mm]
4PB-LP_01	1289	3.63	0.0636
4PB-LP_02	1596	4.49	0.0930
4PB-LP_03	1393	3.92	0.0986
4PB-LP_04	1198	3.37	0.0729
Av	1369	3.85	0.0820
CV	12%	12%	20%

**Table 7:** Results of four-point bending tests (4PB-DIC-LP) in terms of force and deflection under the loading points (recorded by both cantilever transducer, $\eta^{CE}$, and DIC, $\eta^{DIC}$) at peak load P and flexural stress $\sigma_{f}$ according to the classical mono-modulus beam model.

	P [N]	$\sigma_{f}$ [MPa]	$\eta^{\mathrm{CE}}$ [mm]	$\eta^{\mathrm{DIC}}$ [mm]	Av η
					(CV)
4PB-DIC-LP_01	1066	3.00	0.2033	0.1457	0.1745
					(23%)
4PB-DIC-LP_02	914	2.57	0.1107	0.1105	0.1106
					(0.1%)
4PB-DIC-LP_03	875	2.46	0.1166	0.1158	0.1162
					(0.5%)
Av	952	2.68	0.1435	0.1240	
CV	11%	11%	36%	15%	

**Table 8:** Results of the mono-modulus (MM) and bi-modulus model (EB for Euler–Bernoulli and TM for Timoshenko beam model) applied to three-point (3PB-SP and IF-SPN) and four-point (4PB-LP) bending tests using $n=E_{t}/E_{c}=0.52$. The flexural stress value $\sigma_{f}$, vertical displacement of the load application point $\eta_{exp}$, the vertical displacement retrieved by bi-modulus models $\eta_{BM}$, the percentage difference between the displacements $\delta_{BM}$, the vertical displacement retrieved by mono-modulus model $\eta_{MM}$, the percentage difference between the displacements $\delta_{MM}$, the maximum values of the tensile and compressive stress $\sigma_{t}$$\sigma_{c}$ and the position of the neutral axis h-z${}_{c}$ are reported for each bending test.

	$\sigma_{f}$	$\eta_{\exp}$	$\eta_{\mathrm{BM}}$	$\delta_{\mathrm{BM}}$	$\eta_{\mathrm{MM}}$	$\delta_{\mathrm{MM}}$	$\sigma_{t}$	$\sigma_{c}$	h-z${}_{c}$
		(CV)							
	[MPa]	[mm]	[mm]	[%]	[mm]	[%]	[MPa]	[MPa]	[mm]
3PB-SP-MM	4.67	0.1068			0.0139	87%			
		(26%)							
3PB-SP-EB			0.0190	82%			4.01	5.59	23.28
3PB-SP-TM			0.0199	81%			4.01	5.59	23.28
4PB-LP-MM	3.85	0.0820			0.0412	50%			
		(20%)							
4PB-LP-EB			0.0570	31%			3.31	4.60	23.28
4PB-LP-TM			0.0590	28%			3.31	4.60	23.28
IF-SPN-MM	0.78	0.0137			0.0025	82%			
		(31%)							
IF-SPN-EB			0.0047	66%			0.67	0.94	15.95
IF-SPN-TM			0.0053	61%			0.67	0.94	15.95

**Table 9:** Results of the mono-modulus (MM) and bi-modulus model (EB for Euler–Bernoulli and TM for Timoshenko beam model) applied to three-point (3PB-SP and IF-SPN) and four-point (4PB-LP) bending tests employing the average values of the vertical displacements $\eta_{exp}$ of each bending test. The flexural stress value $\sigma_{f}$, the flexural elastic modulus $E_{f}$, the moduli ratio coefficient *n*, the maximum values of the tensile and compressive stress $\sigma_{t}$$\sigma_{c}$, the position of the neutral axis h-z${}_{c}$, the elastic modulus in tension $E_{t}$ and the percentage difference between the evaluated $E_{t}$ and the experimental value of modulus in tension (7422 MPa) $\delta_{E_{t}}$ are reported for each bending test.

	$\sigma_{f}$	$E_{f}$	*n*	$\sigma_{t}$	$\sigma_{c}$	h-z${}_{c}$	$E_{t}$	$\delta_{E_{t}}$
	(CV)	(CV)						
	[MPa]	[MPa]		[MPa]	[MPa]	[mm]	[MPa]	[%]
3PB-SP-MM	4.67	1918						
	(8%)	(24%)						
3PB-SP-EB			0.052	2.87	12.84	32.65	751	90%
3PB-SP-TM			0.063	2.92	11.91	32.06	910	88%
4PB-LP-MM	3.85	7172						
	(12%)	(15%)						
4PB-LP-EB			0.314	3.00	5.42	25.72	4518	39%
4PB-LP-TM			0.348	3.05	5.25	25.25	5002	32%
IF-SPN-MM	0.78	3861						
	(7%)	(28%)						
IF-SPN-EB			0.122	0.52	1.57	20.43	1702	77%
IF-SPN-TM			0.139	0.53	1.49	20.03	1953	74%

## Data Availability

Not applicable.
